# In vivo monitoring of dihydroartemisinin-piperaquine sensitivity in *Plasmodium falciparum* along the China-Myanmar border of Yunnan Province, China from 2007 to 2013

**DOI:** 10.1186/s12936-015-0584-8

**Published:** 2015-02-05

**Authors:** Hui Liu, Heng-lin Yang, Lin-hua Tang, Xing-liang Li, Fang Huang, Jia-zhi Wang, Chun-fu Li, Heng-ye Wang, Ren-hua Nie, Xiang-rui Guo, Ying-xue Lin, Mei Li, Jian Wang, Jian-wei Xu

**Affiliations:** Yunnan Institute of Parasitic Diseases, Yunnan Provincial Center of Malaria Research, Yunnan Provincial Collaborative Innovation Center for Public Health and Disease Prevention and Control, Yunnan Provincial Key Laboratory of Vector-borne Diseases Control and Research, Puer, 665000 China; National Institute of Parasitic Diseases, Chinese Center for Disease Control and Prevention, Shanghai, 200025 PR China; Tengchong County Center for Disease Control and Prevention, Tengchong, 679100 China; Yangjiang County Center for Disease Control and Prevention, Yingjiang, 679300 China

**Keywords:** *Plasmodium falciparum*, Dihydroartemisinin-piperaquine, In vivo test, Resistance, China-Myanmar border

## Abstract

**Background:**

Artemisinin-based combination therapy (ACT) is the recommended first-line treatment of falciparum malaria in all endemic countries. Artemisinin resistance in *Plasmodium falciparum* has been confirmed in the Greater Mekong subregion (GMS). Dihydroartemisinin-piperaquine (DAPQ) is the most commonly used ACT in China. To understand the DAPQ sensitivity of *P. falciparum*, DAPQ resistance was monitored in vivo along the China-Myanmar border from 2007 to 2013.

**Methods:**

Eligible patients with mono-infections of *P. falciparum* were recruited to this study after obtaining full informed consent. DAPQ tablets for different categories of kg body weight ranges were given once a day for three days. Patients were followed up for 42 days. Polymerase chain reaction (PCR) was conducted to distinguish between re-infection and recrudescence, to confirm the *Plasmodium* species. The data were entered and analysed by the Kaplan-Meier method. Treatment outcome was assessed according to the WHO recommended standards.

**Results:**

243 patients were completed valid follow-up. The fever clearance time (FCT) and asexual parasite clearance times (APCT) were, respectively, 36.5 ± 10.9 and 43.5 ± 11.8 hours, and there was an increasing trend of both FCT (F = 268.41, P < 0.0001) and APCT (F = 88.6, P < 0.0001) from 2007 to 2013. Eight (3.3%, 95% confidence interval, 1.4–6.4%) patients present parasitaemia on day three after medication; however they were spontaneous cure on day four. 241 (99.2%; 95% CI, 97.1–99.9%) of the patients were adequate clinical and parasitological response (ACPR) and the proportions of ACPR had not changed significantly from 2007 to 2013 (X^2^ = 2.81, P = 0.7288).

**Conclusion:**

In terms of efficacy, DAPQ is still an effective treatment for falciparum malaria. DAPQ sensitivity in *P. falciparum* had not significantly changed along the China-Myanmar border of Yunnan Province, China. However more attentions should be given to becoming slower fever and parasite clearance.

## Background

Malaria remains one of the major global public health problems [1]. Intensive efforts in controlling malaria are producing impressive results. The number of malaria cases has fallen by more than half in 40% of malaria endemic countries in the recent decade. Estimates suggest that nearly 750,000 lives have been saved in Africa alone thanks to malaria control measures [2]. Artemisinin-based combination therapy is the recommended first-line treatment of falciparum malaria in all endemic countries. However, the emergence of artemisinin resistance in malaria parasites is threatening malaria control and elimination programmes. Artemisinin resistance in *Plasmodium falciparum* has been identified and confirmed in Cambodia, Thailand, Myanmar, and Vietnam [2-10]. Other suspected foci have been identified in the Greater Mekong Subregion (GMS), but are not yet confirmed. The threat must be taken seriously. Resistance to previous generations of anti-malarials spread rapidly around the world, resulting in an increase in child mortality and untold number of deaths [2]. The artemisinin-based combination therapy (ACT) is the most potent weapon in treating falciparum malaria [11] and no other anti-malarial is available that offers the same level of efficacy and tolerability. In order to contain artemisinin resistance, the World Health Organization (WHO) Global Malaria Programme launched the Global Plan for Artemisinin Resistance Containment. Surveillance of artemisinin sensitivity in *P. falciparum* is one of important components to prevent the emergence of new foci of resistance, as well as to limit the spread of resistance [2].

China has declared a national policy for malaria elimination by 2020 [12,13]. ACT is the first-line therapy for falciparum malaria and dihydroartemisinin-piperaquine (DAPQ) is the most common anti-malarial drug in China [14]. Historically, population movement has contributed to the spread of disease. Failure to consider this factor contributed to failure in malaria eradication campaigns in the 1950s and 1960s [15,16]. Resistance to anti-malarial drugs has often threatened malaria elimination efforts and historically has led to the short-term resurgence of malaria incidences and deaths [17]. With the development of modern transportation, the world is becoming smaller and smaller. China has the largest population in the world and Chinese people move around globally [18], especially to some parts of the GMS where artemisinin resistance has been identified and confirmed. The Yunnan Province is in southwestern China and belongs to the GMS. The Chinese Myanmar border is one of the very few remaining areas of malaria transmission in all of China. In 2013, only 423 malaria cases, 81 (19.1%) *P. falciparum*, were reported along the border in China, and the annual parasite incidence was only 0.9 (95% CI, 0.8–1.0) per ten thousand. Monitoring artemisinin resistance in Yunnan Province would contribute both to malaria elimination in China and in containing global artemisinin resistance. From 2007 to 2013, DAPQ resistance was monitored *in vivo* to determine the dynamics of *P. falciparum* sensitivity to DAPQ on the China- Myanmar border.

## Methods

### Surveillance sites and time

**Figure 1:**
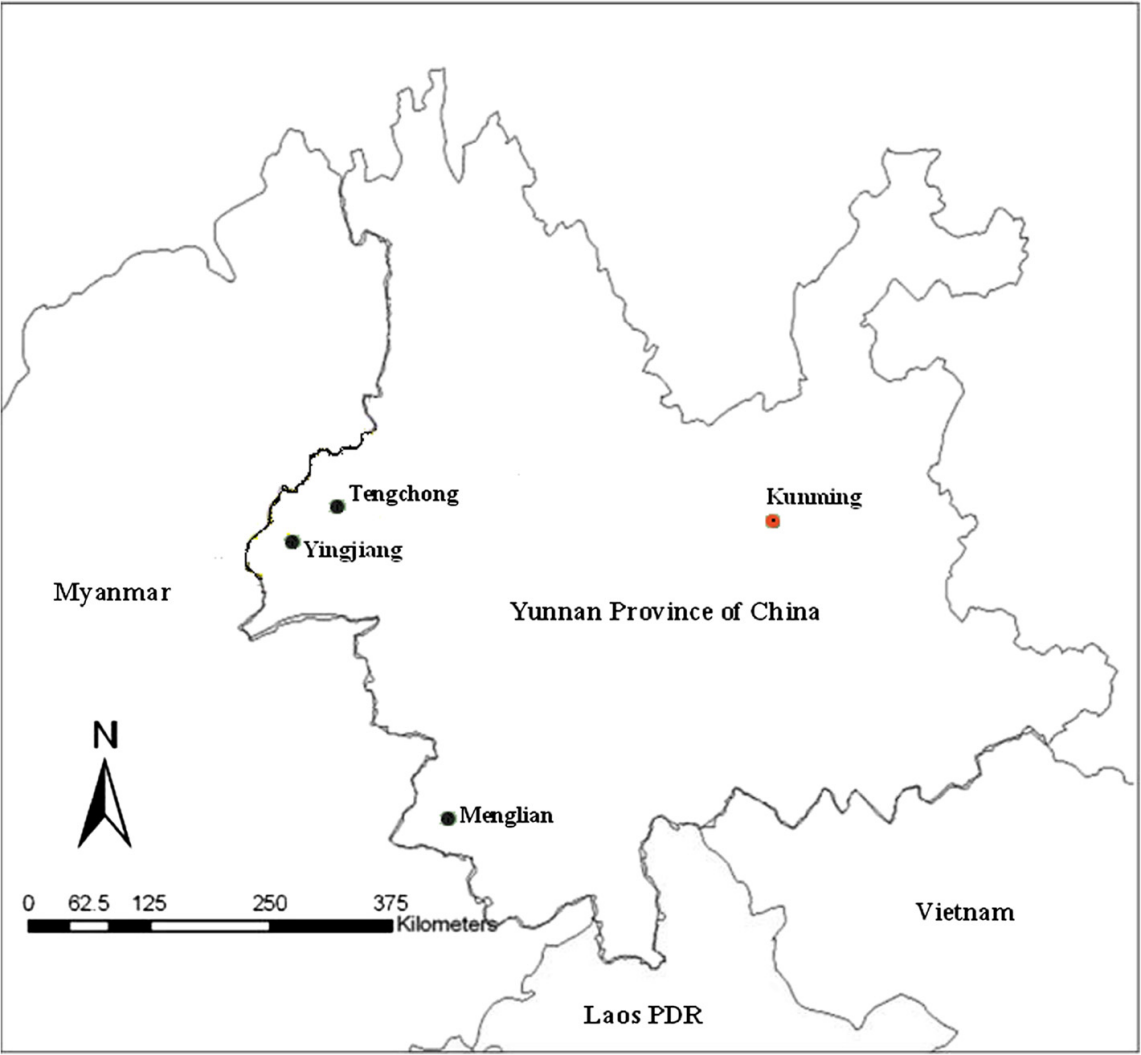
**Surveillance sites: Menglian, Tengchong and Yingjian in Yunnan Province of China and relative to neighbouring countries.**

China and Myanmar share 2,185 kilometre border. There are 19 counties of China and fives special regions of Myanmar on the border. Populations includes 4,687, 896 residents of 19 counties of Yunnan Province of China and 586,000 residents of the five Special Regions of Myanmar. Due to the complex emergency situation in the five Special Regions of Myanmar, where fell outside the coverage of national malaria control efforts supported by Myanmar’s Ministry of Health, effective access of malaria interventions could only be achieved from China. In March, 2008, the baseline survey of evaluation indicator for the sixth grant to China of the Global Fund to fight AIDS, Tuberculosis and Malaria showed that the parasite prevalence rate was 13.6% (761/5585, 95% CI: 12.7–14.6%), and the proportion of *P. falciparum* among slides with parasites was 38.1% (290/761, 95% CI: 34.6–41.7%) in the five Special Regions of Myanmar. In eliminating settings, malaria predominantly occurs in border areas and imported cases tend to represent a majority of recorded cases [19]. In order to recruit malaria patients for the study, surveillance was conducted in Yingjiang of Dehong Prefecture in 2007, Menglian of Puer Prefecture in 2009, in both Tengchong of Baoshan Prefecture and Yingjiang of Dehong Prefecture from 2010 to 2013 (Figure 1).

### Patients and recruiting criteria

Patients whose axillary temperature was ≥ 37.5°C or with a history of fever during the previous 24 hours were diagnosed by using microscopy of thick and thin blood smears. Patients with mono-infections of *P. falciparum* were recruited to this study, after obtaining full informed consent. Only patients older than one year, body weight ≥ 5 kg, presenting with parasite density 500–100,000 parasites per μl were enrolled into the study. Imported malaria was identified as patients who had travelled from endemic areas of Myanmar within one month and were diagnosed as malaria in China [13]. Patients were excluded from the study if any of the following criteria were present: (1) positive pregnancy test or breastfeeding; (2) complicated malaria; (3) having taken any anti-malarial, tetracycline and sulphonamides derivatives drugs within the past seven days; (4) history of hypersensitivity to any of the study drugs; (5) presence of febrile conditions due to diseases other than malaria (e.g. measles, acute lower respiratory tract infection, severe diarrhoea with dehydration) or other known underlying chronic or severe diseases (e.g. cardiac, renal and hepatic diseases, HIV/AIDS); (6) over 60 years; (7) presence of severe malnutrition (defined as a child whose growth standard is below –3 z-score, has symmetrical oedema involving at least the feet or has a mid-upper arm circumference < 110 mm); and, (8) unable to follow-up [20,21].

### Drug and administration

DAPQ was manufactured by Zhejiang Holley Nanhu Pharmaceutical Co. Ltd, and provided by the West Pacific Office of WHO (WPRO/WHO). The drug quality was controlled by WPRO/WHO too. The batch numbers of DAPQ were 600807 (manufactured on 30 Aug 2007), 460909 (23 Sep 2009) and 400911 (20 Sep 2011). Each tablet contains 40 mg base dihydroartemisinin and 320 mg piperaquine phosphate. DAPQ was given once a day for three days and the dosing were based on the recommendation of WPRO/WHO. For convenient administration, the doses were calculated into tablets for different categories of kg body weight ranges (Table 1). The treatment intake was observed while the patients visited the hospital as requested for the first three days. Patients of late clinical failure and late parasitological failure were subsequently given a total dose of artemisinin-naphthoquine 24.5 mg/kg (naphthoquine 7 mg/kg and artemisinin 17.5 mg/kg), once a day for three days [22].

**Table 1:** **Number of DAPQ tablets for each category of body weight range**

**Body weight range (kg)**	**No. tablets, Day 1**	**No. tablets, Day 2**	**No. tablets, Day 3**	**Total Tablets**
5–10	1/2	1/2	1/2	1½
10.1–19	1	1	1	3
19.1–30	1½	1½	1½	4½
30.1–40	2	2	2	6
>40	3	3	3	9

### Laboratory and in vivo clinical monitoring

Parasite microscopy was conducted and axillary temperatures were measured on admission and every 8–12 hours in the first three days. Patients were asked to visit the hospital and further parasitological examinations were performed on day 7, 14, 21, 28, 35 and 42. In a case of patient had not visited the hospital, a researcher actively visited the patient home to prepare blood smears. Malaria blood films were stained with Giemsa, and slides were examined by two independent microscopists and considered negative if no parasites were seen after examination of 200 oil-immersion fields in a thick blood film. Parasite clearance was defined as no asexual parasite per 500 white blood cells being detected in two continuously microscopic examinations with an 8–12 hr interval. Fever clearance was defined as axillary temperatures <37.1°C in duration of 24 hours. Parasites were counted per 500 white blood cells. The number of parasites was calculated as per μl of blood by the level of 8,000 of leukocyte per μl [23]. A filter paper dried blood spot (about 100ul blood) was prepared on admission, day 7, 14, 21, 28, 35 and 42, day of treatment failure or at any other unscheduled visit, and subsequently stored in plastic zip bags containing silica gel dessicant. Polymerase chain reaction (PCR) was performed respectively to distinguish between reinfection and recurrence of asexual parasites of blood stages, to confirm the *Plasmodium* species or to detect mixed infection. DNA extraction and genotype analysis were conducted based on investigation of the three polymorphic genetic markers *msp1*, *msp2*, and *glurp*, according to WHO recommended procedures [24]. Recrudescence was defined as at least one identical allele for each of the three markers in the pre-treatment and post-treatment samples. New infections were diagnosed when all alleles for at least one of the markers differed between the two samples. Cases with new infection were excluded from the analysis [25]. In case of failure after day 7, patients whose PCR results were unknown were excluded from the analysis too [11].

### Classification standards for treatment outcome

Treatment outcome was categorized based on the WHO definitions for early treatment failure (ETF), late clinical failure (LCF), late parasitological failure (LPF), and adequate clinical and parasitological response (ACPR). The ETF definition was to conform to any one of the criteria: (1) danger signs or severe malaria on day 1, 2 or 3, in the presence of parasitaemia; (2) parasitaemia on day 2 higher than on day 0, irrespective of axillary temperature; (3) parasitaemia on day 3 with axillary temperature ≥ 37.5°C; and, (4) parasitaemia on day 3 ≥ 25% of count on day 0. The LCF definition was to satisfy any one of the criteria: (1) danger signs or severe malaria in the presence of parasitaemia on any day between day 4 and day 42 in patients who did not previously meet any of the criteria of ETF; and, (2) presence of parasitaemia on any day between day 4 and day 42 with axillary temperature ≥ 37.5°C in patients who did not previously meet any of the criteria of ETF. LPF definition is to satisfy presence of parasitaemia on any day between day 7 and day 42 with axillary temperature < 37.5°C in patients who did not previously meet any criteria of ETF or LCF. ACPR definition was to satisfy absence of parasitaemia on day 42, irrespective of axillary temperature, in patients who did not previously meet any criteria of ETF, LCF or LPF [20,21].

### Statistical analysis and resistance assessment

The data were by double independent data entry and analysed by the Kaplan-Meier method [22,26]. The patients of loss to follow-up and withdrawal from the study were not involved into the analysis. Mean fever and parasite clearance time were compared by covariance through Epi Info 6.04 [22,26]. The proportions of ETF, LCF, LPF and ACPR were, respectively, compared by Chi-square test for trend of quantitative data [27]. The treatment outcome was assessed on the basis of parasite clearance from the blood.

### Ethical approval

According to the Helsinki Declaration, ethical approval for the study was granted by the Ethics Committee of Yunnan Institute of Parasitic Diseases, China. The purpose of the study was explained and then approval was sought from patients and their caretakers. Informed written consent was obtained from patient or carers of Child patients. All results were kept confidential and were unlinked to any identifying information.

## Results

**Figure 2:**
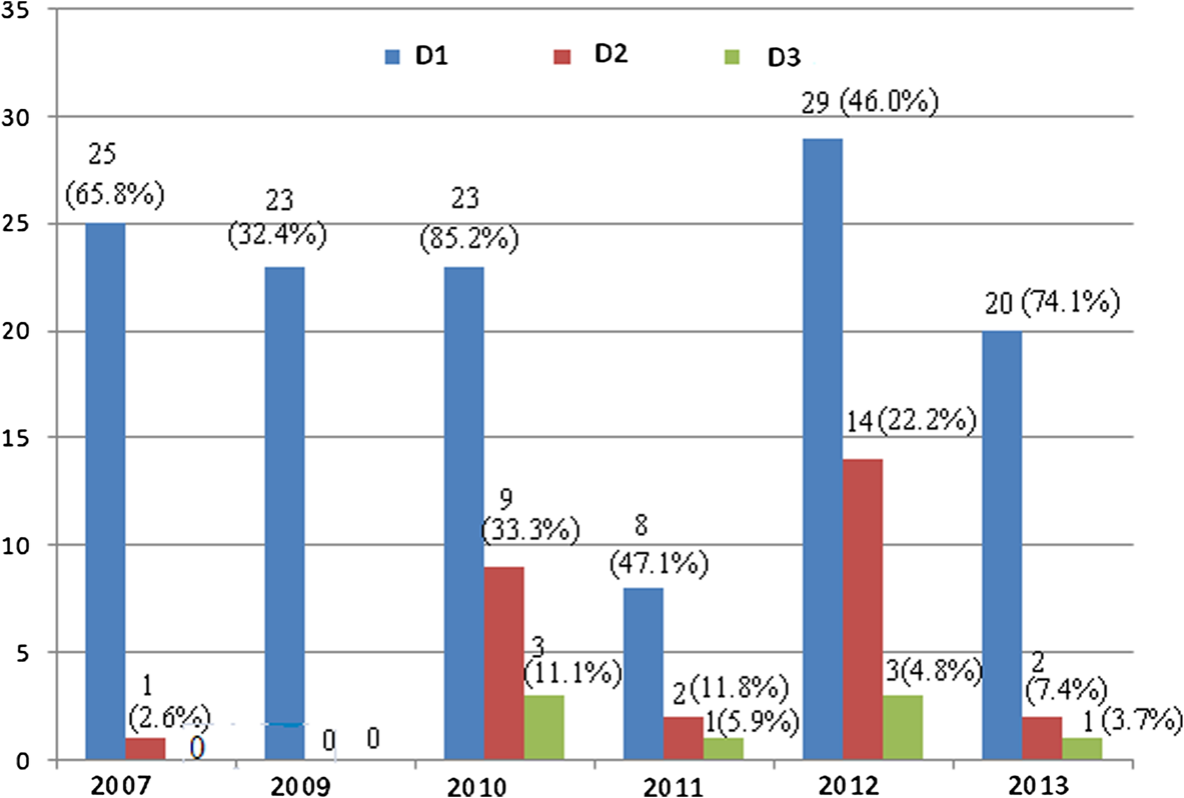
**Number (%) of patients of parasitaemia presence on day 1, 2 and 3 after medication.**

A total of 290 falciparum malaria patients were recruited in the study during 2007–2013 (the study was interrupted in 2008 because of shortage of funding), 22(7.6%) withdrew, 25(8.6%) were lost at follow-up, and 243 (83.8%) completed valid follow-up, of which 178 (72.3%) were Burmese (Table 2). The fever clearance time (FCT) and asexual parasite clearance times (APCT) were, respectively, 36.5 ± 10.9 and 43.5 ± 11.8 hours. The results of covariance analysis showed an increasing trend of both FCT (F = 268.41, P < 0.0001) and APCT (F = 88.6, P < 0.0001) (Table 3). Eight (3.3%, 95% confidence interval, 1.4–6.4%) patients present parasitaemia on day 3 after medication, three in 2010, one in 2011, three in 2012 and one in 2013 (Figure 2); and then parasites were spontaneously cleared by day 4. The results showed that 241 (99.2%; 95% CI, 97.1–99.9%) of the patients were ACPR, one ETF in 2012 and one LCF in 2007, without LPF (Table 3). The results of Chi-square test for trend of quantitative data showed that percentages of ACPR had not changed significantly from 2007 to 2013 (X^2^ = 2.81, P = 0.7288) (Table 3). The ETF patient was cured spontaneously by day four. The LCF patients responded well to the three-day treatment regimen of artemisinin-naphthoquine tablets. The ETF and LCF cases were imported from the neighbouring districts of Myanmar. The PCR identified the LCF as recrudescence on day 28 in 2007, and did not identify any new infection of *P. falciparum.*

**Table 2:** **Baseline characteristics of falciparum malaria patients on the China-Myanmar border, Yunnan Province, China**

	**2007 (n = 38)**	**2009 (n = 71)**	**2010 (n = 27)**	**2011 (n = 17)**	**2012 (n = 63)**	**2013 (n = 27)**	**Total (n = 243)**
Sex							
Male (%)	19 (50)	32 (45.1)	21 (77.8)	17 (100)	44 (69.8)	20 (74.1)	153 (63.0)
Female (%)	19 (50)	39 (54.9)	6 (22.2)	0	19 (30.2)	7 (25.9)	90 (37.0)
Nationality							
Chinese (%)	1(2.6)	10 (14.1)	3 (11.1)	17 (100)	21 (33.3)	13 (48.1)	65 (26.7)
Burmese (%)	37 (97.4)	61 (85.9)	24 (88.9)	0	42 (66.7)	14 (51.9)	178 (72.3)
Age (years)							
Mean (± SD)	16.3 (9.2)	25.0 (3.8)	26.3 (4.2)	33.6 (1.2)	26.1 (8.3)	28.0 (9.6)	25.9 (6.8)
Range	2–60	7–56	2–49	19–48	4–60	2–56	2–60
Body temperature (°C)							
Mean (± SD)	38.3 (0.7)	38.5 (0.6)	38.3 (0.5)	39.3 (0.3)	38.8 (0.9)	38.7 (0.8)	38.6 (0.7)
Rang	37–40.5	37.2–39.4	36.8–40.4	38.0–40.0	36.1–40.4	37.8–39.7	36.1–39.7
Parasite count (per ul)							
Geometric mean	11100	11257	73784	38776	24257	24886	30678
Range (per μl)	760–37120	500–45200	540–821700	5129–119657	596–167132	524–162960	596–821700

**Table 3:** **Treatment responses of falciparum malaria patients on the China-Myanmar border, Yunnan Province, China**

	**2007 (n = 38)**	**2009 (n = 71)**	**2010 (n = 27)**	**2011 (n = 17)**	**2012 (n = 63)**	**2013 (n = 27)**	**Total (n = 243)**	**Statistics**	**P-value**
**FCT (hr)**									
Mean (± SD)	24.5 (8.1)	34.8 (10.4)	36.4 (9.9)	35.3 (9.5)	43.3 (11.4)	45.7 (11.6)	36.5 (10.9)	F = 268.4	<0.0001
Range	16–40	20–48	20–72	22–96	24–96	23–96	16–96		
**50% APCT (hr)**									
Mean (± SD)	24.2 (7.8)	24.6 (8.7)	25.8 (7.4)	26.8 (8.5)	25.9 (8.2)	26.3 (8.4)	25.2 (8.2)	F = 4.14	0.0013
Range	3–29	7–25	8–48	8–48	6–48	7–48	3–48		
**APCT (hr)**									
**Mean (± SD)**	38.4 (7.5)	39.8 (7.9)	41.8 (8.4)	40.9 (9.2)	42.6 (11.3)	52.6 (12.3)	43.5 (11.8)	F = 88.6	<0.0001
**Range**	19–46	16–72	19–72	18–96	16–96	20–72	16–96		
**ETF (%, 95%CI)**	0 (0, 0–9.3)	0 (0, 0–7.0)	0 (0, 0–12.8)	0 (0, 0–19.5)	1 (1.6, 0.04–8.5)	0 (0, 0–12.8)	1 (0.4, 0–2.3)	X^2^ = 2.61	0.7591
**LCF (%, 95%CI)**	1 (2.6, 0.1–13.8)	0 (0, 0–5.1)	0 (0, 0–12.8)	0 (0, 0–19.5)	0 (0, 0–5.7)	0 (0, 0–12.8)	1 (0.4, 0–2.3)	X^2^ = 5.00	0.4164
**LPF (%, 95%CI)**	0 (0, 0–9.3)	0 (0, 0–5.1)	0 (0, 0–12.8)	0 (0, 0–19.5)	0 (0, 0–5.7)	0 (0, 0–12.8)	0 (0, 0–1.5)	-	-
**ACPR (%, 95%CI)**	37 (97.4, 82.7–99.4)	71 (100, 94.9–100)	27 (100, 87.2–100)	17 (100, 80.5–100)	62 (98.4, 91.5–99.9)	27 (100, 87.2–100)	241 (99.2, 97.1–99.9)	X^2^ = 2.81	0.7288

Note: FCT = fever clearance times (hours), APCT = asexual parasite clearance times (hours); ETF = early treatment failure, LCF = late clinical failure, LPF = late parasitological failure, ACPR = adequate clinical and parasitological response, and 95%CI = 95% confidence interval.

## Discussion

*Plasmodium falciparum* resistance to artemisinin derivatives in Southeast Asia threatens malaria control and elimination activities worldwide [28,29]. The results of the study showed that the FCT and APCT were increasing from 2007 to 2013 (Table 3); 3.3% patients present parasitaemia on day 3 after medication, but were spontaneously cured by day 4 without treatment. The principal pharmacodynamic advantage of using artemisinins is that they accelerate parasite clearance by clearing young, circulating, ring-stage parasites and preventing the further maturation and sequestration of these parasites. This effect accounts for the rapidity of the therapeutic response, its lifesaving benefit in patients with severe malaria, and the notable gametocytocidal activity of the drugs [3,28-30]. The reduced susceptibility of ring-stage parasites causes the slow parasite clearance [29-33]. The markedly prolonged time to parasite clearance showed a decline in the efficacy of DAPQ during the six years. However, DAPQ was still highly efficacious for the treatment of falciparum malaria on the China-Myanmar border areas, presumably because of the efficacy of piperaquine phosphate. 99.2% of the patients were ACPR and the proportions of ACPR had not changed significantly from 2007 to 2013 (X^2^ = 2.81, P = 0.7288).

DAPQ sensitivity of *P. falciparum* had been monitored on the China-Myanmar border. Sun *et al.* reported that DAPQ was efficacious for falciparum malaria treatment in 2006 [34], however Liu *et al.* reported a 97.0% of cumulative success rate (CSR) and reduced DAPQ sensitivity in *P. falciparum* of a trial in which the only eight tablets of DAPQ (a dosage for >40 kg body weight) were used for uncomplicated falciparum malaria treatment [35]. In the study by Liu *et al.,* the technical limitation was to not use PCR to distinguish between re-infection and recrudescence, and they conduct their study in Wa State of Myanmar, where *P falciparum* prevalence was high during 2007–2008. The technical limitation and the different regimen might result in the declined CSR, and geographic differences might be one of explanations for reported differences too. Huang *et al.* reported a 95.9% (47/49) of artesunate CSR and a high frequency of mutations in *pfcrt, pfdhfr* and *pfdhps* associated with chloroquine and sulphadoxine–pyrimethamine resistance and no *pfatp6* mutation in *P. falciparum* [36]. Wang *et al.* reported low levels of point mutations in *pfmdr1* and *pfatp6* prevalence and no *pfmdr1* gene amplification detected [37]. The results of the two molecular epidemiology studies showed lack of molecular basis of artemisinin resistance along China-Myanmar border. All these investigations together were not enough to confirm the artemisinin resistance in *P. falciparum* along the China-Myanmar border.

Several limitations should be considered while using the results of study. Firstly, both total and yearly sample size were limited by the difficulty in recruiting patients because of low malaria incidence in eliminating settings, only total 1011 falciparum malaria cases were reported during 2010–2013. Secondly, the surveillance activity was interrupted in 2008 because of shortage of funding. Thirdly, the surveillance sites had to be changed in order to recruit falciparum malaria patients, in Yingjiang in 2007, Menglian in 2009, in both Tengchong and Yingjiang from 2010 to 2013, however all the three sites are on the China-Myanmar border. Fourthly, the study did not use any pharmacokinetics (PK) measurement to ascertain drug absorption and to characterize the concentration–time profile of drugs and the relevant covariates (e.g. immunity, young age and drug interactions), so it did not exclude confounding effect of some variables that could influence APCT [38]. Fifthly, mutations of *PF3D7_1343700* kelch propeller domain (K13-propeller) are important determinants of artemisinin resistance [29]; the study did not investigate mutant K13-propeller alleles. According to the new working definition of ACT or artesunate monotherapy resistance, the suspected resistance is as evidenced by either ≥5% K13-propeller domain mutants or ≥10% of patients who are parasitaemic on day three; and the confirmed resistance should satisfy both ≥5% K13-propeller domain mutants and ≥10% of patients with parasitaemia on day three, or by the persistence of parasites for seven days, or by the presence of parasites on day three and recrudescence within 28/42 days [29,39]. The monitored results showed that only 3.3% (8/243) of patients presented parasitaemia by day three and only 0.8% (2/243) were ETF or LCF. According to the new working definition DAPQ was still sensitive in *P. falciparum* along the China-Myanmar border.

While artemisinin resistance was found on the western border of Cambodia-Thailand and the western border of Thailand-Myanmar [40], *P. falciparum* was still sensitive to artemisinins on the China-Myanmar border. The genetic diversity of malaria parasites and multiclonal infections are correlated with transmission intensity as well as the spread of anti-malarial resistance. Despite the intensified control efforts and the decline of malaria prevalence along China-Myanmar border, the parasite population size and transmission intensity remained high enough to allow effective genetic recombination of the parasites and continued maintenance of genetic diversity in China-Myanmar border area [41]. Therefore, surveillance for artemisinin resistance in *P. falciparum* should be maintained and containment activities should be further strengthened to monitor the spread of resistance, define therapeutic and operational strategies to understand its molecular basis and counter its impact [28,42]. In terms of monitoring artemisinin resistance, treatment failure of ACT is more direct and accurate [43], so it may be more useful in artemisinin sensitivity surveillance.

Detection of drug efficacy is only the first step to producing accessible and useful information for decision makers. The translation of increased access to data on health outcomes into usable evidence for rational policy and planning requires a global coordination and communication effort [44]. In terms of containment measures, a 97.5% of DAPQ CSR for *P. falciparum* treatment had been reported in Hainan Province of China in 2004 [45] and then 100% in 2008 [46], *P. falciparum* has been eliminated by using the strategy of early diagnosis and treatment (EDT) with ACT [47]. The emergence of resistance to artesunate in *P. falciparum*, the strategy of EDT with ACT has reduced malaria in the migrant population living on the Thai-Myanmar border [48]. The addition of one dose of primaquine to ACT could help to counter the spread of artemisinin resistance [49]. Despite the 8-aminoquinoline compound cause patients with G6PD deficiency haemolysis, it can sterilize gametocytes of *P. falciparum.* Lower doses of the gametocytocide would be safer, might still be very effective for blocking transmission.

## Conclusion

In terms of efficacy, DAPQ is still effective for falciparum malaria treatment. DAPQ sensitivity in *P. falciparum* had not significantly changed along the China-Myanmar border of Yunnan Province, China. However more attentions should be given to becoming slower fever and parasite clearance, surveillance for artemisinin resistance in *P. falciparum* should be maintained and containment measures are urgently needed.
